# Sequence-specific ^1^H, ^13^C and ^15^N backbone resonance assignments of the plakin repeat domain of human envoplakin

**DOI:** 10.1007/s12104-015-9659-2

**Published:** 2015-11-21

**Authors:** Mark Jeeves, Claudia Fogl, Caezar Al-Jassar, Martyn Chidgey, Michael Overduin

**Affiliations:** Henry Wellcome Building for Biomolecular NMR Spectroscopy, University of Birmingham, Edgbaston, Birmingham, B15 2TT UK; MRC Laboratory of Molecular Biology, Francis Crick Avenue, Cambridge, CB2 0QH UK; School of Cancer Sciences, University of Birmingham, Edgbaston, Birmingham, B15 2TT UK; Department of Biochemistry, Faculty of Medicine and Dentistry, University of Alberta, 474 Medical Sciences Building, Edmonton, AB T6G 2H7 Canada

**Keywords:** Envoplakin, Plakin repeat domain, Cornified envelope, Plakin, Cytoskeleton, Backbone resonance assignment

## Abstract

The plakin repeat domain is a distinctive hallmark of the plakin superfamily of proteins, which are found within all epithelial tissues. Plakin repeat domains mediate the interactions of these proteins with the cell cytoskeleton and are critical for the maintenance of tissue integrity. Despite their biological importance, no solution state resonance assignments are available for any homologue. Here we report the essentially complete ^1^H, ^13^C and ^15^N backbone chemical shift assignments of the singular 22 kDa plakin repeat domain of human envoplakin, providing the means to investigate its interactions with ligands including intermediate filaments.

## Biological context

Envoplakin is a member of the plakin family of cytolinkers. Plakin proteins connect elements of the cell cytoskeleton to each other and to junctional complexes at the cell membrane. The plakin proteins play a vital role in the maintenance of tissue integrity, particularly in tissues such as the heart and skin that are subjected to high levels of mechanical stress (Al-Jassar et al. 2013). The plakin superfamily consists of seven proteins in mammals, namely envoplakin, periplakin, desmoplakin, plectin, bullous pemphigoid antigen 1 (encoded by the dystonin gene), microtubule-actin cross-linking factor 1 and epiplakin. These proteins are widely expressed and have diverse modular structures but all are characterized by the presence of either a plakin domain, a plakin repeat domain (PRD), or both (Sonnenberg and Liem 2007; Bouameur et al. 2014).

Envoplakin is a 2033 amino acid protein which forms heterodimers with periplakin to initiate formation of the cornified envelope (DiColandrea et al. 2000, Candi et al. 2005). The cornified envelope forms beneath the plasma membrane of keratinocytes during the later stages of epidermal differentiation and is a core component of the epidermal permeability barrier in eukaryotes, preventing water loss and excluding foreign substances and organisms from the environment. In differentiating keratinocytes envoplakin is localized to the plasma membrane, forming contacts with desmosomes, the interdesmosomal membrane and the cell cytoskeleton (Ruhrberg et al. 1996). Envoplakin is targeted by autoantibodies in paraneoplastic pemphigus (Zimmermann et al. 2010), an autoimmune skin blistering condition that accompanies malignant and benign neoplasia, although the role of these antibodies in the aetiology of the disease remains to be determined.

Of the various plakin proteins envoplakin is most closely related to the desmosomal protein desmoplakin. They both contain an N-terminal plakin domain, followed by a central coiled-coil rod domain and a C-terminal tail domain. Three PRD domain subclasses have been designated; types A, B and C. The envoplakin tail domain has a C-type PRD which is joined to the rod domain by a conserved linker domain. The desmoplakin tail domain has one of each type of PRD, in the order A, B, C, with the linker domain joining PRDs B and C. PRD domains mediate direct connections with the cell cytoskeleton that are essential for tissue integrity. Mutations causing premature truncation of the desmoplakin protein and loss of intermediate filament binding cause lethal acantholytic epidemolysis bullosa, a condition that is characterized by catastrophic fluid loss and early death (Jonkman et al. 2005). Single point mutations in PRDs B and C of desmoplakin have been linked to arrhythmogenic right ventricular dysplasia, a heart muscle disorder causing arrhythmia and sudden cardiac death (Alcalai et al. 2003; Yu et al. 2008). To begin understanding the mechanisms, crystal structures have been determined of desmoplakin PRD-B and PRD-C (PDB: ILM7 and ILM5, Choi et al. 2002) and envoplakin PRD-C (4QMD) modules in the absence of ligands. Herein, we report the backbone resonance assignments for a 193 residue protein representing wild-type human envoplakin PRD-C. This facilitates the study of the PRD solution structure, dynamics and interactions with ligands including diverse intermediate filament proteins and binding motifs, and analysis of the mechanism of PRD proteins in tissue formation and disease progression.

## Methods and experimental

A construct comprising the complete human envoplakin PRD module (residues 1822–2014) was expressed with a cleavable N-terminal His-tag using the pProEx HTc vector (Life Technologies) in BL21 (DE3) *E. coli* cells. Cells were grown in M9 media supplemented with ^15^NH_4_Cl and ^13^C-glucose at 37 °C until an OD_600_ of 0.6 was reached. The temperature was then reduced to 18 °C and protein expression induced by the addition of 1 mM isopropyl-β-D-thiogalactopyranoside. Cells were grown for a further 16 h, harvested by centrifugation (7000 g for 15 min) and resuspended in phosphate buffered saline with complete EDTA-free protease inhibitors (Roche). The cells were lysed using an EmulsiFlex-C3 (Avestin) and the lysate cleared by centrifugation (75,000 g for 45 min). The envoplakin PRD was purified from the supernatant by nickel affinity chromatography using HisTrap HP columns (GE Life Sciences). The poly-His tag was cleaved using Tobacco Etch Virus protease, leaving 8 exogenous residues. The PRD was further purified by size exclusion chromatography using a Superdex-75 column (GE Life Sciences).

NMR experiments were performed at 298 K on Agilent NMR spectrometers equipped with cryogenic Z-axis pulse field gradient probes. Backbone assignments were made using BEST versions of the HNCA, HNCACB, HNCOCA, HNCO and HNCACO experiments (Schanda et al. 2006) and a standard CBCACONH pulse sequence (Grzesiek and Bax 1992). The HNCA, HNCACB, HNCO and HNCOCA experiments were performed on a 900 MHz spectrometer while HNCACO and CBCACONH experiments were collected on a 600 MHz spectrometer. Spectra were processed using NMRPipe (Delaglio et al. 1995) and analysed using CCPN software (Vranken et al. 2005).

## Extent of assignments and data deposition

**Fig. 1:**
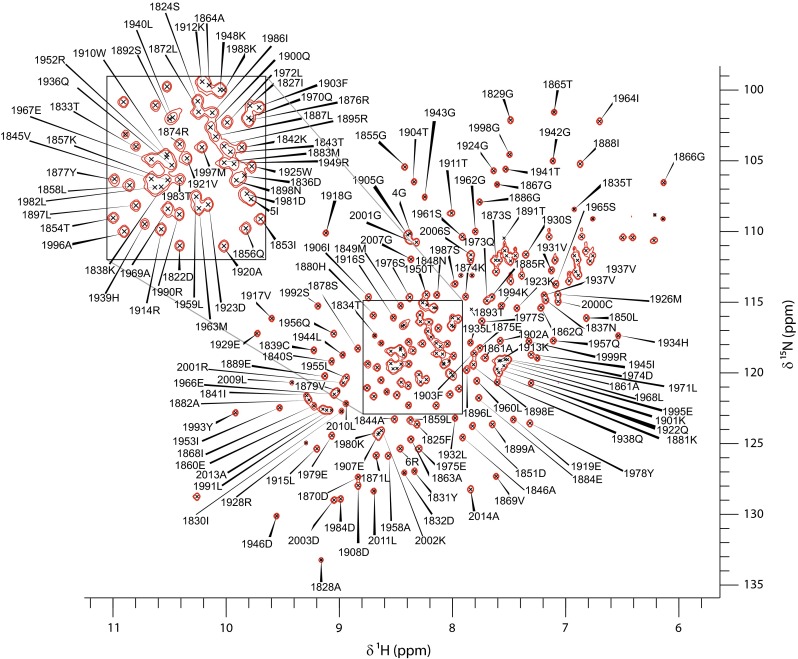
The ^1^H,^15^N-HSQC spectrum of the human envoplakin plakin repeat domain in 50 mM HEPES, 50 mM NaCl, 0.5 mM TCEP, pH 7. Data was collected at 298 K on a Varian 800 MHz spectrometer. Backbone ^1^H,^15^N peaks are labelled with their residue assignments. An expanded section of the central, overlapped region of the spectrum is shown

The ^1^H, ^15^N HSQC of the envoplakin PRD is shown (Fig. 1). Backbone assignments have been completed for 96 % of amide groups, 95 % of C′, 94 % of Cα and 93 % of Cβ non-proline residues. The C′, Cα and Cβ have been determined for all of the proline residues. Assignments for residues 1–3, 7 and 8 of the artefactual remnants of the N-terminal His-tag are missing, and those peaks which are assigned for this element exhibited sharp NMR signals, indicative of disorder. The resonance assignments of Asp1823, Phe1825, Thr 1854, Gln1900, Val1910, Ile1955, Asn1890, Thr1893 and Gln1894 are incomplete. Analysis using TALOS+ (Shen et al. 2009) indicated that Asp1823, Asn1890, Val1910 and Phe1925 are in unstructured elements, Thr1893, Gln1894 and Gln1900 are found in the fourth helix of the protein, and Ile1955 is located in the eighth helix of the domain. Although the latter four residues are in a structured region of the PRD, according to TALOS+ and structure comparison with the known desmoplakin PRD structures (ILM5 and ILM7, Choi et al. 2002) and the crystal structure of the envoplakin PRD (4QMD), the repetitive nature of the 4.5 plakin repeat motifs that define the PRD fold creates difficulties in resolving the highly overlapped chemical shifts of these particular residues (Fig. 1). The chemical shift values for the ^1^H, ^13^C and ^15^N resonances of envoplakin PRD have been deposited at the BioMagResBank (http://www.bmrb.wisc.edu) under accession number 26642.
